# The Relationship between Body Composition, Fatty Acid Metabolism and Diet in Spinal Muscular Atrophy

**DOI:** 10.3390/brainsci11020131

**Published:** 2021-01-20

**Authors:** Katherine S. Watson, Imane Boukhloufi, Melissa Bowerman, Simon H. Parson

**Affiliations:** 1 Institute of Medical Sciences, University of Aberdeen, Aberdeen AB25 2ZD, UK; katherine.s.watson.12@aberdeen.ac.uk; 2 School of Medicine, Keele University, Staffordshire ST5 5BG, UK; imane.boukhloufi@gmail.com; 3 Wolfson Centre for Inherited Neuromuscular Disease, RJAH Orthopaedic Hospital, Oswestry SY10 7AG, UK; 4 Euan MacDonald Centre for Motor Neurone Disease Research, University of Edinburgh, Edinburgh EH16 4SB, UK

**Keywords:** spinal muscular atrophy, survival motor neuron, fatty acid metabolism, nutrition, diet

## Abstract

Spinal muscular atrophy (SMA) is an autosomal recessive condition that results in pathological deficiency of the survival motor neuron (SMN) protein. SMA most frequently presents itself within the first few months of life and is characterized by progressive muscle weakness. As a neuromuscular condition, it prominently affects spinal cord motor neurons and the skeletal muscle they innervate. However, over the past few decades, the SMA phenotype has expanded to include pathologies outside of the neuromuscular system. The current therapeutic SMA landscape is at a turning point, whereby a holistic multi-systemic approach to the understanding of disease pathophysiology is at the forefront of fundamental research and translational endeavours. In particular, there has recently been a renewed interest in body composition and metabolism in SMA patients, specifically that of fatty acids. Indeed, there is increasing evidence of aberrant fat distribution and fatty acid metabolism dysfunction in SMA patients and animal models. This review will explore fatty acid metabolic defects in SMA and discuss how dietary interventions could potentially be used to modulate and reduce the adverse health impacts of these perturbations in SMA patients.

## 1. Introduction

Spinal muscular atrophy (SMA) is a debilitating neuromuscular disorder, which predominantly affects children within the first months of life, affecting 1:10,000 live births [1]. SMA primarily impacts alpha motor neurons in the spinal cord, leading to their death, resulting in muscle wasting, hypotonia and hyporeflexia [2].

**Figure 1:**
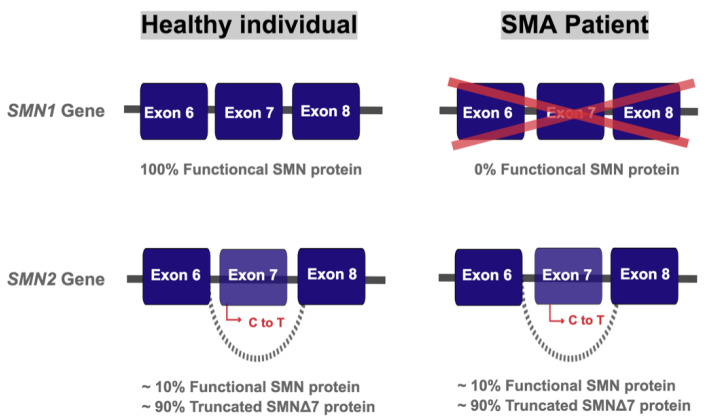
Genetics of spinal muscular atrophy (SMA). The *SMN1* gene produces 100% full-length functional protein. The homologous *SMN2* gene produces about 10% of full-length functional SMN protein and 90% of a truncated and rapidly degraded SMNΔ7 protein, due to a C to T substitution in exon 7. Healthy individuals retain copies of both *SMN1* and *SMN2* while SMA patients have a loss of *SMN1* due to mutations and/or deletions.

SMA pathology is caused by a deficiency in the survival motor neuron (SMN) protein. In >90% of SMA patients, there is homozygous loss of the gene encoding this protein: *survival motor neuron 1* (*SMN1*) [3,4]. This gene was identified in 1995 on the telomeric region of chromosome 5 [5]. Interestingly, this stretch of genome contains a preserved inverted duplication mutation such that there is a near identical gene located closer to the centromere, which is named *SMN2* [4]. This is a unique feature and in some ways represents the existential cause of SMA, as without *SMN2*, humans would not live to develop the condition. There are only five nucleotides that are different between the paralogues, which does not alter coding for amino acids but does affect pre-mRNA splicing [6]. The substitution of a C for a T in the 6+ position of exon 7 of the *SMN2* gene [7] primarily results in the loss of a splicing enhancer, SF2/ASF [8], leading to the skipping of exon 7 in around 90% of transcripts. The truncated protein (SMNΔ7) produced is unstable and less functional compared to the full-length version [9], but is not considered a negative dominant. The remaining 10% of transcripts are structurally and functionally normal. *SMN2* is extremely important in the context of SMA as gene copy number is a positive modifier of the condition. Indeed, it has been shown that disease severity is inversely proportional to the number of *SMN2* copies [10]. In essence, this means that the loss of *SMN1* is compensated for, to a greater or lesser extent, by the number of *SMN2* copies a person possesses and thus the amount of SMN that can still be produced. SMA, therefore, results from the complete loss of *SMN1* and the concurrent retention of at least one copy of *SMN2* (Figure 1).

SMA is clinically classified into types based on severity and age of onset, whereby *SMN2* gene copy number is the major disease modifier, as a greater number of *SMN2* copies results in a larger amount of functional full-length SMN protein being produced [11]. Type I, also known as Werdnig-Hoffman disease, is the most severe and common form of the condition, accounting for 50% of cases [2]. Type I presents at <6 months of age and these babies are unable to sit up independently, the disease usually leading to a premature death before they reach their second birthday [12]. Type II is an intermediate form of the disease with an onset between 6 and 18 months and a reduced lifespan [13]. Patients with this form do not gain the ability to walk and even though they are usually unable to stand without support, some can stand independently [2]. However, these motor aptitudes are not necessarily maintained as although gain of functions are observed, over time, there is a net loss of function for all SMA patients [14]. Type III SMA occurs from 18 months onwards [15]. Type III children will at some point be able to walk and have a normal life expectancy, though with varying degrees of disability [16]. Type IV SMA is an adult onset classification of the disease, whereby patients begin to exhibit muscle weakness between their 30s and 40s, retain the ability to walk and have a typical lifespan [2,17]. Type IV is the rarest form of SMA and accounts for <5% of cases [18]. In general, type I patients have 1–3 *SMN2* copies, type II patients 2–4 copies, type III patients 2–5 copies and type IV patients 4–6 copies [19]. The overlap of copy number between clinically distinct phenotypes does however suggest the presence of additional non-SMN modifiers [19].

SMN is ubiquitously expressed [20] and the cellular machinery it is involved with is crucial to the working of all cell types [21]. Previously, the rhetoric behind SMA focused on why only motor neurons are affected by the loss of a protein that is present in every cell. Over the past few decades, the picture of SMA has been changing, with expanding recognition of non-neurological pathologies that can be attributed to SMN deficiency [22]. Of these, fatty acid oxidation metabolism defects have frequently been reported in both earlier case reports and more recent studies in SMA animal models and patients. Fatty acids have many important functions in the body as they are a principal source of energy [23]. Indeed, fatty acids are crucial components of the human body, whereby they have structural, functional and biological roles such as being an important constituent of the cell membrane as well as acting as a crucial energy source for the heart, skeletal muscle and kidney [23]. Their metabolism also produces a significant amount of adenosine triphosphate (ATP), not only in the postabsorptive and fasted states when glucose supply is limited but also when glucose is abundantly available [23]. In this review, we will provide an up-to-date assessment of fatty acid metabolism defects in SMA as well as discuss the interactions between body composition, nutrition and metabolism in relation to SMA pathology.

## 2. Body Composition of SMA Patients

By conventional body mass index (BMI) measurements, SMA patients often appear normal or underweight [24]. However, when subject to more rigorous anthropometric testing, there appears to be a recurring incidence of increased adiposity within the SMA population. Indeed, in a study of 34 type I patients, aged between 1 and 36 months, a significant increased fat mass compared to age-matched controls was observed, despite 35% of the SMA cohort meeting the criteria for failure to thrive [25]. Similar findings were reported when comparing type I and II children [24], whereby the mean BMI z score (BMI adjusted for age) was −6.3 and −2.1 for SMA type I and type II respectively, values that would be interpreted as distinctly undernourished. According to their BMI, none of the children with type I and II SMA were overweight or obese, yet in comparison to age and sex-matched children, they had on average 20% more fat [24]. An additional study on type II and III patients concluded that while they displayed a significant reduction of lean mass, aligned with the muscle wasting that typifies SMA, they also had significant elevation in absolute fat mass [26]. Indeed, in the study group of 25 children, 10 patients (40%) had a fat mass index (FMI) >85th percentile that indicates “at risk of overweight” and 5 patients (20%) had an FMI >90th percentile indicating “overweight”. This is in accordance with the Centre for Disease Control and Prevention (CDC)’s weight classifications. These findings highlight the fact that BMI testing is not a sufficient tool to estimate adiposity in SMA patients and that for the most part, there is an inverse relationship between the BMI and FMI of SMA patients.

One possible contributor to the increased fat mass in SMA patients is an imbalance between dietary intake and energy expenditure. The degenerative nature of SMA makes this scenario quite plausible as progression of the disease leads to increasing immobilisation. In 2015, a retrospective study was conducted based on clinical data from 60 SMA patients (types I, II and III) in order to assess nutrient intake and establish nutritional status [27]. Anthropometric data was analysed from two visits made 2 years apart on average. At initial assessment, 9 children (15%) were considered to be malnourished, based on weight for age z scores (WAZ) out with ±2. However, there was no discrimination between malnourishment due to overfeeding (WAZ >+2) and malnourishment due to underfeeding (WAZ <−2). In the subgroup of 17 device-fed patients, 6 (35%) were considered optimally fed, 5 (30%) overfed and 6 (35%) underfed. There is therefore little clarity regarding the nutritional requirements for SMA patients as there are cases of both under and overfeeding. It is additionally worth noting that the methods in this study included using the “Schofield equation” to estimate basal metabolic rate, which has been shown to overestimate resting energy expenditure (REE) by between 11% [24] and ~19% [28] compared to REE measured by indirect calorimetry. Thus, SMA patients reported as being underfed may in fact not have been and the incidence of overfeeding may indeed be larger than reported. This could have particular significance with regard to the reported increased fat mass in SMA, as lack of accuracy surrounding assessments directly limits the ability to make appropriate dietary recommendations, which may lead to energy imbalance. Additionally, energy requirements based on the Schofield equation were used on the cohort as a whole, as it was assumed that activity levels would not vary much between patients. However, as described above, motor capabilities within types I-III SMA patients can in fact vary quite extensively [29]. Furthermore, in SMA patients, caloric intake has been shown to correlate with motor skills measured by the Hammersmith Motor Scale, and there may be differences between SMA subtypes based on function and ambulation [26]. Thus, the increased fat mass prevalent within SMA children may be due to a lack of understanding of how best to manage dietary requirements. However, it is also worth considering whether increased fat mass could be due to mechanistic defects in fat metabolism, specifically fatty acid oxidation, which could have a significant repercussion on whole-body homeostasis and health of SMA patients.

## 3. Fatty Acid Oxidation and Metabolism

### 3.1. Basics of Fatty Acid Oxidation and Metabolism

Fats have multiple roles within the body, from membrane components to signalling molecules, but they are most commonly associated with energy metabolism. In the form of triglycerides, they are the primary source of energy storage as they contain a higher energy content per gram (9 kcal/g) than both proteins and carbohydrates (4 kcal/g) [30]. In the fasted state, adipose tissue releases triglycerides (fatty acids and glycerol) for utilisation in energy production [31]. Glycerol can be converted to produce glucose and fatty acids are progressively broken down via oxidation, of which there are three types (alpha, beta and omega), to ultimately form acetyl-CoA. Both glucose and acetyl-CoA then enter the citric acid cycle [32].

**Figure 2:**
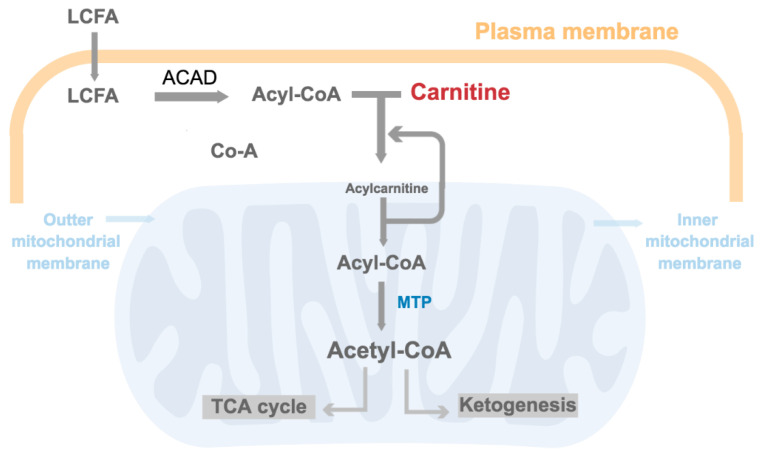
Schematic diagram of mitochondrial fatty acid entry and beta oxidation. Long chain fatty acid (LCFA) is activated by bonding with CoA upon cell entry. Acyl-CoA dehydrogenase (ACAD) catalyzes this first reaction. This molecule, now an acyl-CoA, is shuttled through the mitochondrial membrane by forming an ester bond with carnitine, thus generating acylcarnitine. Once inside the mitochondria, acylcarnitine is broken down to produce free carnitine, which can be recycled to shuttle in further fatty acids in the mitochondria. The long chain acyl-CoA generated is then oxidised to produce acetyl-CoA, a process catalysed by mitochondrial trifunctional protein (MTP). Acetyl-CoA can be utilised in the tricarboxylic acid cycle (TCA) cycle or participate in ketogenesis.

Oxidation of fatty acids predominantly occurs by beta oxidation in the matrix of mitochondria, following activation of fatty acids through thioester bonding with Co-enzyme A (CoA). This process removes a hydroxyl group from the fatty acids, forming highly polar thioesters called acyl-CoA molecules. Long chain acyl-CoA molecules (10+ carbons) cannot freely pass through mitochondrial membranes and require shuttling in the form of carnitine derivatives, referred to as acylcarnitines or esterified carnitines, whereby an ester bond links the fatty acyl-CoA to the carnitine molecule [33]. Once inside the mitochondria, fatty acids follow the beta oxidation pathway, which essentially comprises a repeating sequence of 4 reactions catalysed by acyl-CoA dehydrogenase, enoyl-CoA hydratase, hydroxy acyl-CoA dehydrogenase, and ketoacyl-CoA thiolase, respectively, cleaving 2 carbons from the acyl chain each time to produce acetyl-CoA (Figure 2).

**Figure 3:**
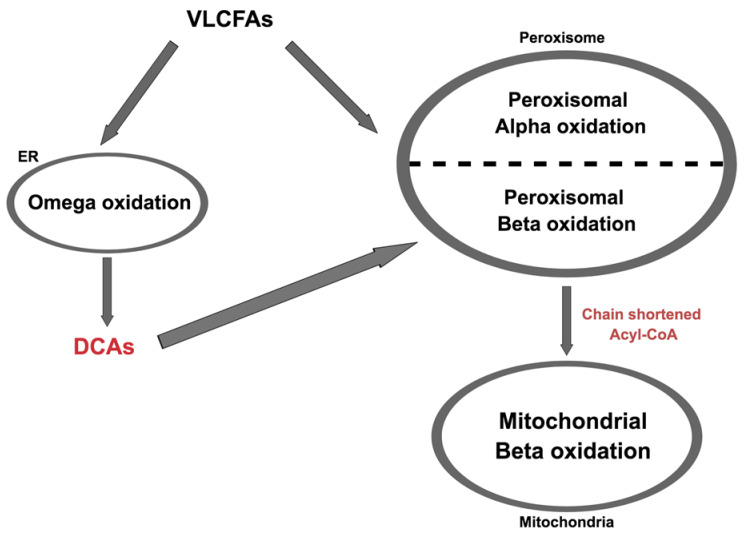
Schematic diagram of alpha and omega oxidation. Very long chain fatty acids (VLCFAs) either go through peroxisomal alpha oxidation or omega oxidation in the endoplasmic reticulum (ER). The latter produces dicarboxylic acids (DCAs) that undergo peroxisomal beta oxidation, produce shortened acyl-CoAs that then go through mitochondrial beta oxidation.

While the majority of beta oxidation takes place in the mitochondria, additional beta oxidation also occurs inside peroxisomes. Peroxisomes are double membrane-bound organelles found in eukaryotic cells and they are responsible for the catabolism of various types of molecules as well as the exclusive site of alpha oxidation of fatty acids [34] that otherwise cannot directly undergo beta oxidation. Alpha oxidation cleaves only one carbon at a time and is utilised for the breakdown of 3-methyl branched fatty acids [35] (Figure 3). For very long chained fatty acids (VLCFAs, >22 carbons), omega oxidation that occurs in the endoplasmic reticulum (ER), converts these molecules into dicarboxylic acids (DCAs), which can then undergo beta oxidation in the peroxisome [36] (Figure 3). Omega oxidation accounts for only a small proportion of all fatty acid oxidations [37] but compensates when there is defective/deficient beta oxidation, leading to an accumulation of DCAs, the excess of which is excreted into the urine. DCAs are an important, though often non-specific marker for fatty acid oxidation disorders [38].

### 3.2. Fatty Acid Oxidation and Metabolism Defects in SMA

One of the earliest accounts associating SMA with potential fatty acid metabolic abnormalities is a case report by Kelley and Sladky in 1986 [39], where a type II male hospitalised for respiratory distress was noted to excrete abnormally high levels of medium chain DCAs in proportion to 3- hydroxybutyrate (BHB). BHB is a ketone body that, alongside DCAs, is normally produced in states such as fasting or catabolic illnesses when there is decrease in glucose levels in the blood and subsequent increase in circulating fatty acids [40].

Fatty acid metabolism defects have since been regularly reported in SMA such as in a study of 14 SMA patients that displayed dicarboxylic aciduria, high excretion of urinary acylcarnitines and carnitine deficiency in both muscle and serum [41]. A deficiency in acyl-CoA dehydrogenase, the enzyme responsible for the first step of beta oxidation, was also noted. Subsequent studies helped to provide additional mechanistic insight into fatty acid metabolism defects and dicarboxylic aciduria in SMA children [42]. Indeed, the aciduria of small and medium chained fatty acids was most evident in SMA type I patients compared to SMA type II and III patients, suggesting that metabolic abnormalities vary with SMA severity [42]. In this same study, five children (2 type I, 2 type II and 1 type III) provided muscle biopsies for further fatty acid oxidation analyses [42]. Specifically, the activity of 5 key enzymes involved in fatty acid metabolism regulation (long-chain 3-hydroxyacyl-CoA dehydrogenase (LCHAD), short-chain 3-hydroxyacyl-CoA dehydrogenase (SCHAD), 3- ketothiolase, acetoacetyl-CoA thiolase and enoyl-CoA hydratase) was analysed. All of these enzymes are involved in beta oxidation except for acetoacetyl-CoA thiolase, which is implicated in the production of ketone bodies. Interestingly, in all 5 patients, the activity of enoyl-CoA hydratase was normal, while all other enzymatic activity was significantly reduced. Free and acylcarnitine levels were also assessed and significant elevations in the percentage of esterified carnitine (i.e., fatty acyl-CoA-carnitine ester bonds) were observed in SMA patients, the highest of which occurred in the youngest type I and type II children [42], suggesting aberrant fatty acid oxidation processes in SMA patients. However, in this study, it is unclear where the normal range of values originated from and from which state (fasting or fed). In addition, the time elapsed between the patient eating and the test being performed was not mentioned. However, in a subsequent study, increased acylcarnitine levels were also observed in SMA patients (>50% esterified) compared to normal values of 10–25% (fed) and 30–50% (fasting) [43].

Fasting and non-fasting fatty acid profiles revealed similar abnormalities in serum and urine samples from 50 SMA patients when compared to healthy controls and infants suffering from a denervating condition that is not caused by loss of SMN [44]. The SMA patients were categorised into “severe infantile” and “juvenile chronic”, which presumably refers to type I and types II/III, respectively. In the fasted state, when perturbations are most likely to become evident, significantly higher levels of dodecanoic acid (C12), saturated medium-chain fatty acid with a 12-carbon chain, were found in severe SMA patients compared to the other groups, including the defined “disease control” and “normal control” groups, who demonstrated the typical and expected increase of C12 in the plasma of fasted healthy children [45]. When glucose levels fall, such as in fasting states, there is an expected switch from glucose to fatty acid metabolism [46]. A build-up of C12 suggests that there is either a block of further breakdown past 12 carbons by the LCHAD/trifunctional enzyme complex or that transport of these long chain fatty acids into the mitochondrial matrix is impaired. Whereas longer chained fatty acids will undergo carnitine esterification at high concentrations and shorter chained fatty acids are able to diffuse into cells, C12 chain length is such that it will not readily do either and so remains in the serum [47]. Furthermore, urinary samples from these SMA patients showed increases in non-specific DCAs. In fact, the ratio of DCA:ketones in infantile SMA is strikingly similar to that of patients with known fatty acid oxidation defects [44]. Additionally, in this study, there is supporting evidence for the hypothesis that metabolic dysfunction reflects SMA severity as the DCA:ketone ratios for all the severe SMA children under 10 months old were decidedly abnormal while type II infants of a similar age had ratios that were well within the normal limits [44]. Three infantile SMA patients in this study who survived to 32, 54, and 63 months were tested a second time. Interestingly, the C12 levels recorded at the older age were lower than the first measurements and similar to the range in control groups, suggesting an age-dependent influence on metabolic perturbations, perhaps due to developmental functions of SMN.

**Figure 4:**
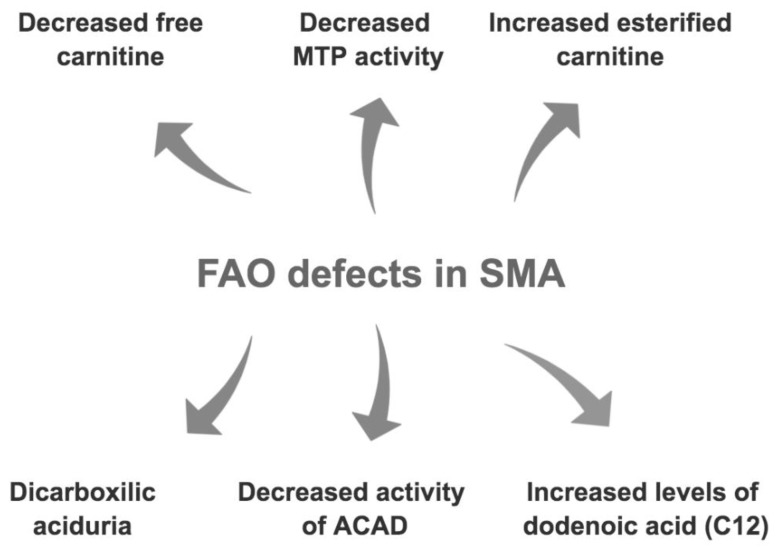
Summary of fatty acid oxidation (FAO) defects in SMA. Mitochondrial trifunctional protein (MTP), acyl carnitine dehydrogenase (ACAD).

There is therefore consistent evidence of disruption of fatty acid metabolism in SMA patients (Figure 4). What is not totally clear, however, is the cause of these metabolic perturbations. Whereas increased fat mass in SMA children points towards the need to better quantify a recommended dietary intake, the prospect of fatty acid oxidation defects suggests that the components of that diet may also need to be more carefully considered in SMA.

## 4. Nutrition and Fatty Acid Metabolism in SMA

### 4.1. Dietary Approaches Evaluated in SMA Mouse Models

As discussed above, there is a need to better understand the relationship between fatty acid metabolism defects, aberrant fat accumulation and diet in SMA. SMA mouse models are well established, display the canonical hallmarks of the disease and have been invaluable to our understanding of pathological mechanisms and therapeutic development [48]. Notably, SMA mouse models of varying severities and genetic backgrounds display significant fatty acid metabolism defects similar to those reported in SMA patients such as microvesicular steatosis, high levels of triglycerides and long chain fatty acids length alterations [49].

The importance of nutrition and its impact on SMA disease progression was first highlighted following the observation that severe SMA mice lived 2–4 days longer when maintained on a 9% fat diet compared to a 5.2% fat diet [50]. Interestingly, as SMA mice die before weaning age, the benefits of the dietary interventions were dependent on being transmitted by the maternal milk. As part of the SMA phenotype in mice includes poorer feeding, the improvement in survival achieved by the higher fat diet may be because SMA mice achieved a greater calorific intake for less effort. In humans, however, some studies have found no direct association between maternal diet and fat content of milk [51] while others suggest some inter-relations that are perhaps dependent on the stage of milk production (i.e., colostrum vs transitional milk vs mature milk) [52].

Both 9% and 5.2% fat-containing diets were found to have no effect on spinal cord motor neuron loss, onset of body mass loss or overall body weight. There were however differences noted when looking at energy metabolites. In general, wild type (WT) mice had higher blood glucose levels than SMA mice. The 5.2% fat diet-fed mice (both WT and SMA) were found to have higher blood glucose than 9% fat diet-fed mice. Additionally, ketone levels were higher in SMA mice compared to WT animals and this was most evident in SMA mice fed the 5.2% fat diet. These results suggest that some dietary component is affecting the SMA phenotype, which may be fat content but also additional dietary elements that differed between both diets (e.g., protein, individual amino acids, fatty acids).

In a more recent study, a high-fat diet (HFD) (60% Kcal fat) and low-fat diet (LFD) (10% Kcal fat) were compared to normal chow (NC) in SMA mice [53]. Similar to above, the diets were administered to the dams until the pups could freely feed on their own. However, it is unknown whether the full extent of the nutritional content of each diet was carried in the milk of the dams to the pups. Nevertheless, introduction of the LFD doubled the life expectancy of SMA mice in comparison to normal chow, while HFD did not lead to any improvements. Interestingly, weight, Smn levels and hepatic fat content were only changed to a limited extent regardless of diet. In addition, liver damage was reduced in SMA mice fed the LFD compared to those receiving NC, suggesting that LFD may restore appropriate proportions of energy substrates by reducing circulating fats and increasing glucose. More importantly, LFD led to reduced plasma ketones, implying reduced beta-oxidation and a lower reliance on fatty acids as an energy source. From these results, it appears that diet modulation aimed at decreasing fatty substrate availability diminishes the load on fatty acid oxidation processes and ameliorates overall health.

The contradictory conclusions between both studies, whereby one favours a HFD [50] and the other a LFD [53], makes it hard to conclude which type of diet should be used for SMA. The opposing results may be from the individual dietary compositions within the diets, variability in fat content used, disease severity of mouse models and/or genetic backgrounds. Therefore, further investigations are required to fully appreciate which dietary components are key to improving fatty acid metabolism and overall health in SMA.

While diet alone can have a profound effect on the health of an organism, in the context of medical care, it is also very important to consider its interaction with a drug treatment. Indeed, the bioavailability and pharmacokinetics of pharmacological compounds are especially influenced by diet and body composition [54,55]. D158944, a member of the quinazoline family, which promotes inclusion of exon 7 in *SMN2* transcripts, was administered to mice either on a Picolab20 or Harlan diet [50]. A 15% increase in lifespan was observed in mice treated with D158944 and mice fed the PicoLab20 diet lived longer than those reared on Harlan. Interestingly, D158944 levels were 32% higher in the brains of mice fed the PicoLab20 diet compared to those on the Harlan diet, suggesting that diet can impact the bioavailability of the drug levels in the central nervous system (CNS).

A further example of drug and diet interaction in SMA mice is demonstrated in a study evaluating the therapeutic potential of Trichostatin (TSA), a histone deacetylase inhibitor [56]. This drug itself had proven promising by lengthening the mean lifespan of SMA mice. When combined with “aggressive nutritional support”, there was a 170% increase in survival of these SMA mice compared to the ones lacking the drug combination [57]. In addition, combining TSA and nutritional support improved the motor skills of SMA mice compared to animals only receiving nutritional support.

Overall, while these studies in pre-clinical models clearly demonstrate a role for dietary interventions in the management of SMA symptoms and disease progression, there remains a lack of clear identification of the key specific dietary constituents and their levels. A better understanding of these components is essential for the development of nutritional guidelines for SMA patients [58].

### 4.2. Dietary Interventions in SMA Patients

The current nutritional guidelines for SMA patients recommend dietary adjustments based on assessments of swallowing, dysphagia, weight, gastrointestinal function, glycaemic control and bone health [59] but not on fatty acid metabolism defects.

However, patients and their families have nevertheless developed and adopted elemental diets. Elemental diets are amino acid based and low in fat feeds, meant to provide nutrition in the simplest elements to reduce the energy cost of digestion [60]. Diets such as these are thought to be beneficial in the background of poor digestion as they provide an easily digestible protein source and a low-fat content aids gastric motility and reduces reflux [61]. Around half of the type 1 respondents to a nutritional survey reported gastric reflux and formula tolerance issues including gastrointestinal pain [62], making an elemental diet an attractive option. Indeed, between two visits made on average 18 months apart, the percentage of type I patients on an elemental diet increased from 38% to 68%, as reported in an observational study of caloric and nutrient intake [25]. Interestingly, some components of the elemental diet are similar to those in the LFD evaluated in SMA mice, suggesting that elemental diets could be positively modulating fatty acid defects in SMA patients [53].

It is important to note, however, that diets that are low in fat, such as elemental diets, are at risk of being deficient in essential fatty acids and may require particular attention to ensure an adequate intake of all nutrients and essentials acids [63]. In an observational study of caloric and nutrient intake [25], the number of diets [25] being supplemented with oil (15% at first visit, 38% at the second) is far less than the number using elemental formula (38% at visit 1 and 68% at visit 2). Furthermore, common nutrients at risk of deficiency due to inadequate intake (i.e., lower than recommended daily intake for age) were considered, and it was found that >25% of the cohort where deficient in alpha- linolenic fatty acid, linoleic fatty acid, vitamin A, vitamin D, vitamin E, vitamin K, folate, calcium, iron, and magnesium. Notably, the vitamins (A, D, E, K) listed here are all fat-soluble vitamins. This further suggests that fat intake needs to be carefully managed.

There is thus a clear need for more in-depth pre-clinical and clinical studies on dietary interventions in SMA to help inform clear and evidence-based nutritional guidelines aimed at reducing the impact of aberrant fatty acid metabolism on whole-body health.

## 5. Conclusions

The first account of fatty acid metabolism defects in SMA was in the late 1980s [39]. Since then, additional studies and reports have shed light on the relationship between SMA pathology and fatty acid abnormalities such as carnitine deficiencies, high levels of medium chain DCAs in proportion to BHB, acyl-CoA dehydrogenase deficiency and accumulation of dodecanoic acid C12 [40,41,46]. Notably, these fatty acid defects occur alongside an increased fat mass observed in SMA patients, in contradiction to BMI values and ongoing muscle wasting. Consequently, there is a need to better understand how nutrition and diet modulation (e.g., high-fat, low-fat and elemental diets) can impact SMA disease progression and fatty acid metabolism.

Most tissues are involved in fatty acid metabolism, however, adipose tissue, skeletal muscle and liver are quantitatively more important than others. Each of these tissues has a store of triacylglycerol that can be mobilized in a regulated way to release fatty acids. There is a clear cooperation amongst these tissues and an overlap of fatty acid metabolism pathways in order to maintain whole body homeostasis [46]. Importantly, intrinsic defects in skeletal muscle, liver and adipose tissue have been reported in SMA mice and patients [49,64,65] and their impaired function could cause and/or amplify the fatty acid metabolic defects in SMA. Furthermore, it is possible that loss of SMN may directly impact fatty acid metabolism through its previously reported roles in mRNA splicing [66], actin dynamics [67,68], endocytosis [69], and key metabolic pathways (e.g., glucocorticoid-Krüppel-like 15 [70]), which have all been implicated in regulating fatty acid and lipid metabolism [71,72,73,74,75,76]. Therefore, a more thorough investigation of the intrinsic and extrinsic contributions of each of these tissues to fatty acid metabolism defects in SMA could help elucidate their respective roles.

Whilst the present review focused on fatty acid metabolism, other metabolic pathways have been implicated in SMA pathology. Indeed, glucose metabolism defects such as fasting hyperglycemia, glucose intolerance and hyperglucagonemia have been observed in SMA mice and patients [77,78]. Moreover, perturbed amino acid metabolism has also been reported in SMA mice [70]. Finally, in addition to the strong evidence of disrupted fatty acid metabolism in SMA presented here, recent studies have shown a strong susceptibility for SMA patients to develop dyslipidaemia accompanied with fatty liver disease [49]. Given the inter-relationship between metabolic pathways, it will be essential to understand what causes their dysregulation in SMA and how they may be influenced by each other and nutritional intake.

From the studies presented here, it appears that both the severity of disease and age critically impact fatty acid metabolism defects in SMA patients, which should be taken into consideration when undertaking studies to establish nutritional guidelines. Of importance is that in the current therapeutic landscape of SMN-dependent therapies, patients are living much longer and reaching milestones never before seen [79]. Consequently, not only co-morbidities related to the development of impaired metabolic pathways, but also other perturbations related to puberty and aging should be taken into account when establishing dietary interventions.

There is also significant evidence that, in addition to SMA, fatty acid metabolism may play a role in the pathophysiology of other neurodegenerative and neuromuscular disorders such as amyotrophic lateral sclerosis (ALS) [80], multiple sclerosis, Alzheimer’s disease and Parkinson’s disease [81]. Of particular interest in the present review are the fatty acid metabolism perturbations observed in patients with ALS, a motor neuron disease that shares several pathological similarities with SMA [82]. Alterations in fatty acid and lipid metabolism have been reported in neurons, skeletal muscle and serum of ALS pre-clinical models and patients, impacting energy use, structural integrity and key signaling pathways [80,83,84,85,86]. Notably, a recent genome-wide meta-analysis study found that the *acyl-CoA synthetase long chain family member 5* (*ACSL5*) gene, whose protein regulates lipid and fatty acid metabolism pathways [87,88], was associated with ALS and its accompanying weight loss [89]. As such, several dietary interventions aimed at modulating fatty acid and lipid metabolism abnormalities (e.g., high caloric diet [90,91], ketogenic diet [92] and acetyl-L-carnitine supplementation [93]) have been evaluated in ALS pre-clinical models and patients, with varying success [80]. Therefore, a better understanding of fatty acid metabolism defects and the impact of dietary interventions in other neurodegenerative and neuromuscular disorders could significantly help advance mechanistic insights, therapeutic development and nutritional management for SMA patients.

In conclusion, further work is needed to determine optimal dietary practices to support the quality of life and well-being of SMA patients [58], which can easily complement the SMN-dependent and -independent drug treatments currently approved or being evaluated for the treatment of this devastating neuromuscular disorder [19]. Indeed, future clinical investigations of fatty acid metabolism defects in SMA patients treated with *SMN* gene-based therapies will be essential to better understand the nature of the metabolic perturbations and the contribution of different CNS and peripheral tissues to the fatty acid metabolism defects.

## Data Availability

Data sharing not applicable.
